# Cardiovascular risk factor control in patients with covert brain infarcts in the prospective SILENT cohort study

**DOI:** 10.1093/esj/aakaf006

**Published:** 2026-01-01

**Authors:** Alice de Vautibault, Adnan Mujanovic, Valentin Amar, Laurent Roten, Urs Fischer, Marialuisa Zedde, Rosario Pascarella, Michail Giannakakis, Vasilis Tentoulouris, Elias Auer, Bernhard Siepen, Marta Olive Gadea, Arsany Hakim, Christina Marti, Anna Boronylo, Morin Beyeler, Jean-Philippe Cottier, Grégoire Boulouis, Laurent Fauchier, Anne Bernard, Jérôme Roumy, Thomas Meinel, Marco Pasi

**Affiliations:** Neurology Department, Centre Hospitalier Universitaire de Tours, Université de Tours, Tours, France; Institute of Neuroradiology, Inselspital, Bern University Hospital, University of Bern, Bern, Switzerland; Neurology Department, Centre Hospitalier Universitaire de Tours, Université de Tours, Tours, France; Department of Cardiology, Inselspital, Bern University Hospital, University of Bern, Bern, Switzerland; Department of Neurology, Inselspital, Bern University Hospital, University of Bern, Bern, Switzerland; Azienda Unità Sanitaria Locale-IRCCS di Reggio Emilia, Neurology Unit-Stroke Unit, Reggio Emilia, Italy; Neuroradiology Unit, Ospedale Santa Maria della Misericordia, AULSS5 Polesana, Rovigo, Italy; Department of Neurology, Inselspital, Bern University Hospital, University of Bern, Bern, Switzerland; Department of Neurology, Inselspital, Bern University Hospital, University of Bern, Bern, Switzerland; Department of Neurology, Inselspital, Bern University Hospital, University of Bern, Bern, Switzerland; Graduate School of Health Science, University of Bern, Bern, Switzerland; Department of Neurology, Inselspital, Bern University Hospital, University of Bern, Bern, Switzerland; Institute of Neuroradiology, Inselspital, Bern University Hospital, University of Bern, Bern, Switzerland; Institute of Neuroradiology, Inselspital, Bern University Hospital, University of Bern, Bern, Switzerland; Department of Neurology, Inselspital, Bern University Hospital, University of Bern, Bern, Switzerland; Department of Neurology, Inselspital, Bern University Hospital, University of Bern, Bern, Switzerland; Department of Neurology, Inselspital, Bern University Hospital, University of Bern, Bern, Switzerland; Department of Diagnostic and Interventional Neuroradiology, University Hospital of Tours, Tours, France; Department of Diagnostic and Interventional Neuroradiology, University Hospital of Tours, Tours, France; Department of Cardiology, Tours University Hospital, Tours, France; Department of Cardiology, Tours University Hospital, Tours, France; Medical Imaging Department, Hôpital Bretonneau, CHU Tours, Tours, France; Department of Neurology, Inselspital, Bern University Hospital, University of Bern, Bern, Switzerland; Neurology Department, Centre Hospitalier Universitaire de Tours, Université de Tours, Tours, France

**Keywords:** antiplatelet agent, brain MRI, covert cerebral infarction (CBI), cardiovascular risk factors (cvRFs), high blood pressure (HBP), lacune, silent cohort, statin

## Abstract

**Introduction:**

Covert brain infarctions (CBIs) are associated with cardiovascular risk factors (cvRFs). We aimed to evaluate the presence and therapeutic implications of modifiable cvRFs in patients with incidentally discovered CBI on routine neuroimaging.

**Patients and methods:**

The SILENT cohort (NCT05685069) is a prospective, multicentred European cohort recruiting patients with incidentally detected focal CBIs on routine MRI, without prior clinical stroke. Modifiable cvRFs and their control were assessed using applicable international guidelines during a dedicated outpatient visit, including a clinical examination and laboratory work-up. Associations between cvRF profiles and the number of CBIs were analysed using linear regression.

**Results:**

We included 231 patients (mean age 65 years, *n* = 130 [56%] male) with a total of 445 CBI lesions. Most CBIs were of lacunar type (*n* = 226; 51%) and the most common location was the cerebellum (*n* = 220; 50%). One hundred and fifty (65%) patients had at least 1, 112 (49%) at least 2 and 56 (24%) at least 3 known modifiable cvRFs. Among hypertensive patients, 69 (53%) had uncontrolled hypertension; 22 (65%) of diabetics were insufficiently controlled and 74 (58%) patients with dyslipidaemia had poorly controlled low-density lipoprotein cholesterol. Therapeutic measures were made for 144 patients (62%), including antiplatelet initiation in 107 (46%) and a statin in 69 (30%). The number of cvRFs per patient was significantly associated with the number of CBIs, rate ratio 1.08 (95% Confidence Interval (CI), 1.04−1.13).

**Conclusion:**

In patients with incidentally discovered CBI, we found a high burden of poorly controlled cvRFs. Our findings highlight the importance and yield of a dedicated clinical and laboratory assessment of cvRFs in patients with CBIs.

## Graphical Abstract

**Graphical Abstract:**
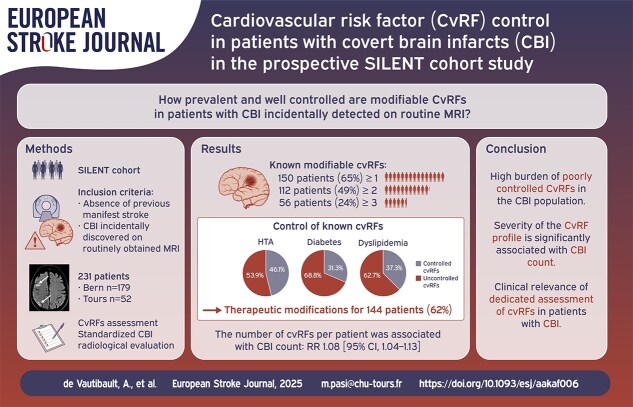

## Introduction

Covert brain infarctions (CBI) are defined as focal presumed ischemic cerebral lesions that are detected by neuroimaging without neurological clinical symptoms suggestive of stroke or transient ischemic attack (TIA).^1–3^ Covert brain infarctions are among the most common incidental finding on brain imaging^4^ with prevalence estimated to be at least of 5% in individuals over 50 years old^5^ and up to 30% in the general elderly population over 70.^6^ In population-based research cohorts, CBIs have been associated with an increased risk of future symptomatic stroke and cognitive decline, and are now considered clinically relevant cerebrovascular disease.^7^ However, a recent survey showed that only 18% of neurologists and neuroradiologists have formal institutional protocols to manage CBI.^8^ As a result, neurology consultation is obtained in <10% of cases for patients with a newly discovered CBI.^9^

In research cohorts, the risk factors profile associated with CBI and symptomatic stroke has been reported to be identical and includes modifiable cardiovascular risk factors (cvRFs) such as hypertension, diabetes mellitus, dyslipidaemia, current smoking, physical inactivity and obesity.^10^ However, compared to manifest ischemic stroke, few studies have evaluated the cvRFs profile and management in individuals with CBIs identified in clinical routine. International guidelines recommend evaluating cvRFs in patients with CBI for primary stroke prevention.^10–12^ The evidence supporting this approach remains weak, partly due to the lack of clinical trials focused specifically on the assessment and management of people with CBI as the primary trial population.

We aimed to describe the risk factor profile of individuals with CBIs in terms of presence, control and new diagnosis. Furthermore, we evaluated therapeutic measures following a dedicated CBIs outpatient visit. Finally, we investigated whether the number of cvRFs was associated with the number of CBIs.

## Patients and methods

The SILENT cohort study (NCT05685069) is an ongoing, prospective, observational, bicentric European study initiated by the Department of Neurology of the University of Bern (Switzerland) in 2019, with the Department of Neurology of Tours University Hospital (France) joining in February 2023. The study protocol was approved by the Swiss Ethics Committees on 13 February 2019 In Switzerland. All participants provided written, informed consent at baseline consultation. In France, after oral and written information, patients could object to data use, leading to exclusion. Inclusion was limited to recent CBI findings, with the timing depending on the local workflow.

Patient recruitment was primarily conducted through the neuroradiology department, using a dedicated informational sheet displayed in the interpretation room. This sheet included definitions and imaging examples of the different lesion types considered as CBIs. Recruitment was done through systematic screening of brain MRIs performed for non-stroke or non-TIA indications, and referrals from neurologists or other specialists. All imaging studies were initially analysed locally by one of the neuroradiologists from our departments. Neurological care for enrolled patients included a baseline consultation and a comprehensive etiological stroke work-up. This assessment comprised non-invasive angiography of cervical and intracranial arteries, transthoracic echocardiography, minimum 24-hour electrocardiogram monitoring and blood tests including glucose levels, glycated haemoglobin (HbA1c), liver function, lipid profile, cardiac markers, blood count, electrolytes, renal function and inflammation markers.

### Inclusion criteria

The main inclusion criterion was no clinical history of a manifest stroke or TIA and incidental CBI, deemed clearly chronic ischemic lesions on routine brain MRI, without corresponding focal neurological signs attributable to the lesion. Stroke-free status was assessed using a stroke-free status questionnaire, which was conducted by the neurologist during consultation.^13^^,^^14^ This questionnaire was retrospective, based on the clinician’s observations and the patient’s own report, enquiring more specifically about potential lesion-specific symptoms. Patients were excluded if they were younger than 18 years old or if the projected life expectancy was <2 years. Exclusion criteria were a contraindication to MRI or to any of the etiologic examinations of stroke. Covert neurological deficits were allowed, meaning that subtle deficits (eg, mild cognitive, mood or gait changes) could be identified on detailed neurological examination and standardised tools (Montreal Cognitive Assessment, EuroQol-5 Dimension and Beck Depression Inventory) but were not reported by the patient as stroke events. Patients with significant cognitive decline or dementia affecting autonomy were excluded, provided only those with capacity to understand and adhere to the proposed management were included.

### Imaging assessment

All patients received an MRI at 1.5 or 3 tesla, including at least DWI, T1-weighted sequences (T1W), T2 (T2W) and/or fluid-attenuated inversion recovery (FLAIR), susceptibility-weighted imaging (SWI) or T2 gradient echo. Definition and identification of CBIs on MRI were based on current consensus literature^4^^,^^15–18^: (1) DWI-positive lesions defined as restricted diffusion (high DWI signal and low apparent diffusion coefficient value) lesion occurring in either white or grey matter, located in the cerebrum, cerebellum or brain stem; (2) Cavitatory lesions (ie, lacunar type) ≥ 3 mm in size with cerebrospinal fluid signal on all sequences that are slit or wedge shaped with an irregular margin and not longitudinally aligned with perforating vessels or with a multiple, bilateral symmetrical distribution; (3) T2W hyperintense/T1W hypointense lesions defined as a focal lesion present (1) within cortical grey matter or deep grey matter; (2) in the white matter, which are discontinuous with the classic confluent periventricular T2 intense change of leukoaraiosis (Fazekas ≥2) and without a significant patient history of severe trauma, radiation, drug toxicity or carbon monoxide poisoning. For all CBIs, the location was also reported as: supratentorial subcortical or cortical, basal ganglia, cerebellar or brainstem.

### Clinical data collection

Demographic data were collected during baseline consultation. These included sex, age, personal and family history of cardiovascular disease, known cvRFs (hypertension, diabetes, dyslipidemia, smoking status, pack-years, body mass index (BMI), history of obstructive sleep apnea syndrome (OSAS), CHA2DS2-VA Score,^19^ and current treatments including antiplatelets (acetylsalicylic acid, clopidogrel, ticagrelor), anticoagulants (direct oral anticoagulants, vitamin K antagonists and heparins). A clinical neurological examination and vital signs assessment were also performed.

### Definitions of cvRFs

The presence and control of modifiable cvRFs were assessed according to international guidelines for hypertension, dyslipidaemia, diabetes, smoking and obesity.^20–25^ In line with these criteria, modifiable cvRFs were classified as known (documented medical history or specific treatment), uncontrolled (according to recommended targets, irrespective of prior history) or previously undiagnosed (first identified at consultation in patients without prior history). High blood pressure (HBP) was diagnosed if blood pressure was ≥140/90 mmHg during the consultation. When available, HBP was also defined by home monitoring with an average of > 135/85 mmHg from 3 daily measurements over 3 days or from a 24-hour recording, which, represents the most reliable approach.^26^

Poorly controlled low-density lipoprotein (LDL) cholesterol was defined according to individual target levels: patients without history of ischemic heart disease or atherosclerosis had a treatment target LDL cholesterol (LDLc) < 2.6 mmol/L. Those with a history of atherosclerosis had a target LDLc < 1.8 mmol/L, while patients with history of ischemic heart disease or peripheral artery events had a target < 1.4 mmol/L.

Diabetes was diagnosed by HbA1c > 6.5%, blood glucose > 2 g/L or fasting blood glucose > 1.26 g/L on 2 occasions. The treatment target for diabetic patients was HbA1c < 7%.

Active smoking was defined as consuming ≥1 cigarette per day, while overweight and obesity were defined as BMI > 25 kg/m^2^ and > 30 kg/m^2^, respectively. Obstructive sleep apnea syndrome was diagnosed with an apnea-hypopnea index ≥5 with symptoms or ≥15 without symptoms.

Therapeutic measures and medication adjustments based on the assessment of cvRFs were prospectively recorded during neurological outpatient visits, in accordance with primary cardiovascular prevention guidelines.

### Statistical analysis

The descriptive analysis, expressed as absolute numbers and percentages, assessed the risk factor profile of patients with CBIs, focusing on the presence, control status and newly identified diagnoses. We evaluated therapeutic measures following a dedicated CBI outpatient visit. We also compared patients with ≥1 uncontrolled CVRF to the overall cohort using Student’s t-test or Chi-squared test, as appropriate. Statistical significance was defined as a *P* value < .05. Additionally, we assessed the association between the number of CBIs (as the dependent variable) and each specific cvRF and the number of cvRFs using a Poisson regression model analysed both as a continuous variable and in categorical groups (≥1, ≥2 and ≥3 cvRFs). Subgroup analyses were also performed according to CBI location and type to assess potential associations with cvRFs. Model assumptions were verified, confirming absence of overdispersion and satisfaction of the log-link linearity assumption. Results were reported as rate ratios (RRs) with 95% CIs. We conducted a univariable analysis to identify relevant variables (ie, those with a *P* value < .1). Subsequently, we performed a multivariable descriptive regression analysis, adjusting for variables with *P* < .05 in univariable models, as well as age and sex.

No imputation was performed. All analyses were conducted as complete case analyses (ie, participants were included in each model only if they had complete data for the variables involved).

## Results

The SILENT study included 231 patients diagnosed with CBI—179 from Bern University Hospital and 52 from Tours University Hospital. A flow chart of patient screening and inclusion is available in the supplemental materials, Figure S1. The baseline characteristics of the included patients, including the cvRF profile, are described in Table 1. These characteristics were then compared with those of patients with at least 1 uncontrolled cvRF at the time of management. No statistically significant differences were found between the 2 groups. Comparisons of demographics and clinical characteristics between patients recruited in Bern and Tours are shown in the supplemental materials, Table S1. There were no significant differences concerning age, sex and past medical history. MRI indications were vertigo (18%), headache (12%), mild cognitive impairment (8%), seizures (6%), suspected inflammation (2%), trauma (3%), tinnitus (2%), acute neurological deficit (6%) and other reasons (43%). Covert brain infarction count was slightly higher in Tours (2.3 ± 1.5) compared to Bern (1.8 ± 1.2; *P* = .006).

**Table 1:** Demographic and clinical characteristics of the overall cohort and of patients with ≥1 uncontrolled cvRF at management.

Demographics and clinical characteristics		SILENT cohort *n* = 231 (%)	≥ 1 cvRF uncontrolled *n* = 146 (%)
Mean age ± sd—years		65.2 ± 13.7	65.5 ± 13.7
Male sex—nb. (%)		130 (56.3)	80 (54.8)
Number of CBI ± SD		1.9 ± 1.3	1.9 ± 1.2
Past medical history			
	Atrial fibrillation	24 (10.4)	14 (9.6)
	CAD	40 (17.3)	26 (17.8)
	PAD	18 (7.8)	14 (9.6)
	Mean CHA2DS2-VA ± SD	1.8 ± 1.5	1.9 ± 1.5
Cardiovascular risk factor in past medical history			
	Hypertension	131 (56.7)	89 (61)
	Diabetes	34 (14.7)	29 (19.9)
	Dyslipidemia	127 (55)	84 (57.5)
	OSAS	46 (19.9)	29 (19.9)
	Active smoking	46 (19.9)	26 (17.8)
	Obesity	45 (19.5)	29 (19.9)
	At least 1 cvRF	150 (64.9)	96 (65.8)
	At least 2 cvRFs	112 (48.5)	65 (44.5)
	At least 3 cvRFs	56 (24.2)	30 (20.5)
Cardiovascular risk factors profile			
	Mean systolic blood pressure ± SD—mmHg	136.2 ± 20.4	137.4 ± 20.6
	Mean diastolic blood pressure ± SD—mmHg	80.7 ± 12.4	81.2 ± 12.7
	Mean HbA1c ± SD—%	5.8 ± 0.8	5.8 ± 0.8
	Mean fasting glucose ± SD—g/L	1.2 ± 0.8	1.2 ± 1
	LDL ± SD—mmol/L	2.8 ± 1.2	2.8 ± 1.2
Preceding treatments			
	Antiplatelet drugs	107 (46.3)	69 (47.3)
	Anticoagulation drugs	32 (13.9)	18 (12.3)
	Antihypertensive drugs	124 (53.7)	86 (58.9)
	Statin/ezetimibe	103 (44.6)	66 (45.2)
	Oral antidiabetic drugs	26 (11.3)	23 (15.8)

Missing data regarding personal medical histories: CAD (*n* = 5), PAD (*n* = 5), hypertension (*n* = 3), dyslipidaemia (*n* = 22), OSAS (*n* = 25), active smoking (*n* = 2) and obesity (*n* = 7).

Abbreviations: cvRFs = cardiovascular risk factors; CAD = coronary artery disease; CBI = covert brain infarction; HbA1c = glycated haemoglobin; LDL = low density lipoprotein; nb = number; OSAS = obstructive sleep apnea syndrome; PAD = peripheral artery disease.

**Figure 1:**
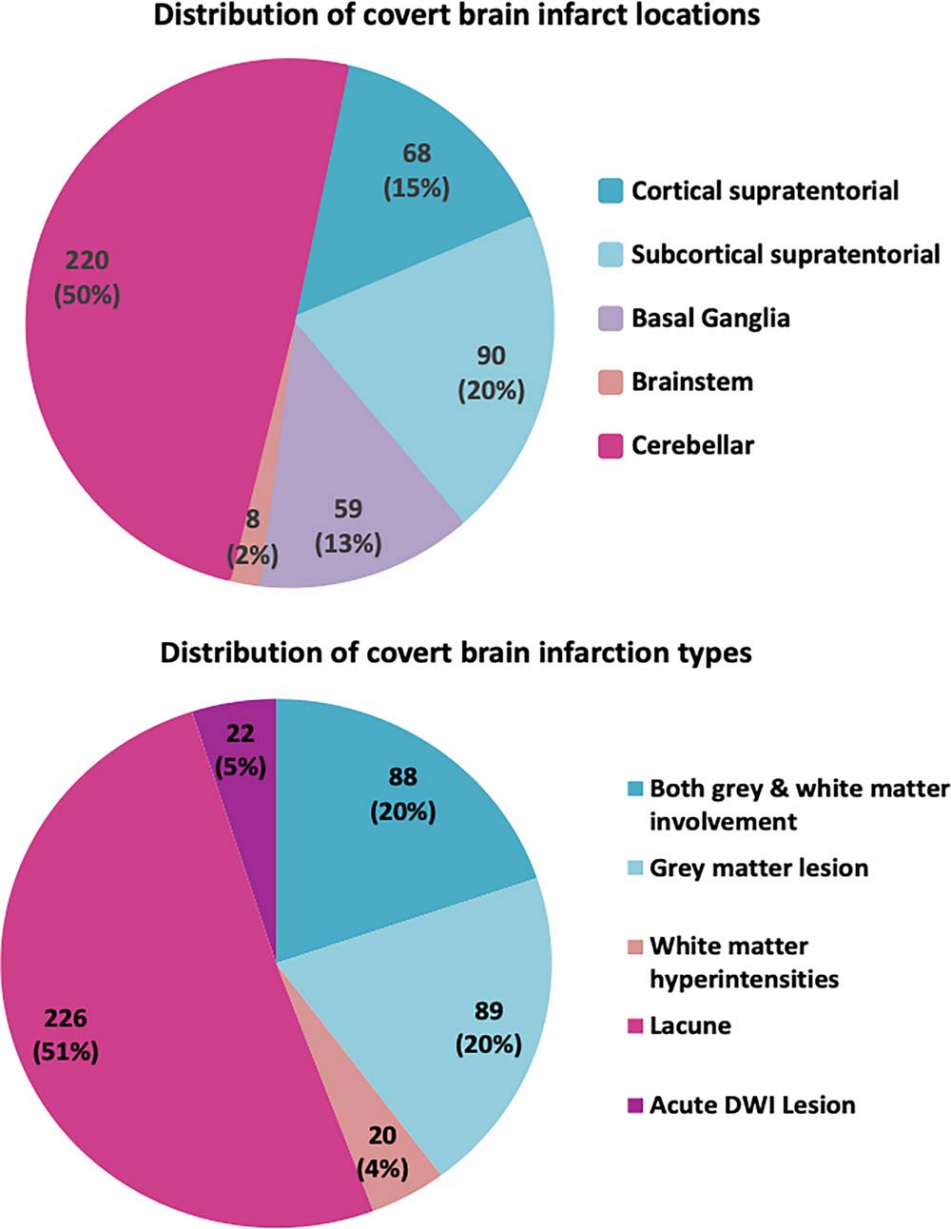
Distribution of CBI by locations and types. Note: CBI = covert brain infarction.

The neuroimaging evaluation found a total of 445 CBIs (range 1—9 CBIs per patient). Sensitivity analyses did not show differences in CBI prevalence between 1.5 T and 3 T MRI, consistent with previous findings from the Bern cohort.^27^ The distribution of CBI count per patient was as follows: 1 (*n* = 119), 2 (*n* = 53), 3 (*n* = 33), 4 (*n* = 13), 5 (*n* = 12) and 9 (*n* = 1). The locations and types of the lesions are reported in Figure 1. Regarding the radiological phenotype classification, we identified 197 T2W hyperintense/T1W hypointense lesions, 226 cavitary lesions (ie, lacunar-type) and 22 acute DWI lesions. Subgroup analyses according to CBI location and type (eg, lacunar versus DWI-positive lesions) were also performed, but no significant associations were observed.

Sixty four percent of patients had at least 1, 48.5% at least 2 and 24.2% at least 3 known modifiable cvRFs. Specifically, among the patients with known hypertension, 69 (52.7%) presented elevated blood pressure at consultation. Among the known diabetic patients, 22 (64.7%) had elevated glycated haemoglobin levels. Furthermore, of the patients with history of dyslipidaemia, 74 (58.3%) had LDL cholesterol levels exceeding their recommended targets, determined by their cardiovascular risk profiles.

During the baseline neurological assessment, among all included patients we identified previously undiagnosed HBP in 29 patients (12.6%), diabetes in 16 patients (6.9%) and dyslipidaemia in 51 patients (22.1%).

Therapeutic modifications were implemented for 144 patients in the cohort (62.3%) with multiple adjustments recommended in 59 (25.5%) of patients. An antiplatelet agent was introduced for 107 patients (46.3%), an antihypertensive drug for 8 patients (3.5%) and a statin for 69 patients (29.9%). About 10 patients (4.3%) received other types of therapeutic measures, such as addition of anti-PCSK9 agents.

The number of known cvRFs per patient was significantly associated with the number of CBI observed on MRI, RR 1.08 (95% CI, 1.04−1.13; Table 2). On univariable (Table 2) and multivariable (Table 3) analyses, the number of CBIs was associated with male sex, HBP and diabetes.

## Discussion

Our study found that patients with CBIs identified in clinical routine, frequently show a poorly treated cvRF profile requiring targeted management according to international guidelines.^10–12^ Over half had hypertension or known dyslipidemia. Among known hypertensive patients, half had poorly controlled blood pressure. Two thirds of known diabetic patients had suboptimal HbA1c levels, and over 60% of dyslipidemic patients had LDL levels above target based on their cardiovascular profiles. These findings are consistent with previous population-based cohorts, which reported up to two-thirds of hypertensive or diabetic individuals remained insufficiently controlled; in the National Health and Nutrition Examination Survey (NHANES), 48% of hypertensive adults achieved target blood pressure, supporting the high burden observed in our CBI cohort.^28–32^ Furthermore, in our study, initial assessment revealed previously undiagnosed cvRFs in 22% of patients. Our results also showed that CBI burden (ie, the number of CBI) was associated with cvRFs presence and severity.

**Table 2:** Rate ratio between number of CBIs for each patient and known cvRF in univariable analysis.

	Risk factor	Rate ratio	Lower CI	Upper CI	*P-*value
Number of CBI	Age	1.01	1.00	1.01	.001
	Male sex	1.16	1.04	1.31	.011
	HBP	1.38	1.22	1.57	<.001
	Diabetes	1.32	1.15	1.52	<.001
	Dyslipidemia	1.06	0.94	1.21	.322
	OSAS	1.00	0.86	1.15	.949
	AF	1.27	1.09	1.48	.003
	At least 1 cvRF	1.35	1.11	1.65	.003
	At least 2 cvRFs	1.23	1.10	1.38	<.001
	At least 3 cvRFs	1.23	1.10	1.38	<.001
	Number of cvRFs	1.08	1.04	1.13	<.001

Abbreviations: AF = atrial fibrillation; CBI = covert brain infarction; cvRF = cardiovascular risk factor; HBP = high blood pressure; OSAS = obstructive sleep apnea syndrome.

**Table 3:** Adjusted rate ratio between number of CBIs and each known cvRF in multivariable analysis.

	Risk factor	Rate ratio	Lower CI	Upper CI	*P-v*alue
Number of CBI	Age	1.00	1.00	1.01	.581
	Male sex	1.17	1.04	1.32	.009
	HBP	1.33	1.15	1.53	<.001
	Diabetes	1.20	1.04	1.39	.014
	AF	1.15	0.98	1.35	.097

Abbreviations: CBI = covert brain infarction; CI = confidence interval; HBP = high blood pressure; AF = atrial fibrillation. Adjusted for variables with *P* < .05 in univariable analysis: age, sex, hypertension, diabetes and atrial fibrillation.

Our study is one of the first attempts that specifically evaluated the cvRF profile specifically in patients with CBIs without an history of manifest stroke. In comparison to our cohort, in a population of ischemic stroke survivors, 99% of patients had at least 1 suboptimally controlled risk factor, 80% had prehypertension or hypertension, 67% were overweight or obese, 60% had suboptimal LDL and 45% had impaired fasting glucose.^33^ Furthermore, in large national cohorts, patients with stroke showed poorer control of key cvRFs compared to those with myocardial infarction, with lower rates of blood pressure control (49% vs 58%), LDL < 100 mg/dL (37% vs 49%) and statin use (58% vs 79%).^34^ These findings highlight the need for improved vascular risk control across the full spectrum of cerebrovascular disease. Regarding the CBI location, we found that half of the lesions were cerebellar, 20% were subcortical supratentorial and 15% were cortical supratentorial. These findings are consistent with those of Vynckier et al., which reported that CBIs in 574 stroke patients were predominantly cerebellar (31%) and subcortical supratentorial (31%), followed by cortical supratentorial involvement (24%).^15^

The ESO guidelines, despite low evidence quality, strongly recommend antihypertensive treatment in hypertensive patients (≥140/90 mm Hg) with covert cerebral small vessel disease, to prevent parenchymal injury progression and related clinical manifestations.^11^ Lipid-lowering and antidiabetic medications should be adjusted according to the cardiovascular risk profile. Statins may be considered in older adults with CBI even without other indications, to prevent the progression of cerebral small vessel disease biomarkers, though clinical efficacy remains unconfirmed.^11^ In line with this statement, a recent systematic review suggested taking 40 mg/day of pravastatin for at least 2 years in patients over 60 years old could reduce CBI incidence.^35^ Our results suggest that modifiable cvRFs are not optimally controlled in a non-negligible proportion of patients with CBI. In this perspective, future studies—including the ongoing SILENT cohort—will assess whether optimised cvRFs control of patients with CBIs reduces cardiovascular events.

Finally, we aimed to evaluate whether CBI burden (ie, the number of CBIs) is associated with the cvRF profile (ie, type of cvRF) and severity (ie, the number of cvRFs). Our findings show that among modifiable cvRFs, hypertension and diabetes were associated with higher CBI burden. Having multiple cvRFs (≥2) was also associated with an increased CBIs number, confirming the association between cvRFs and CBI burden. However, the absence of a comparison group without CBI and the cross-sectional design limits the ability to draw causal inferences from our findings, and should be considered when interpreting these associations*.* In the SILENT longitudinal study, we will specifically assess whether optimised cvRF control reduces CBI progression (ie, incident CBIs on the 2-year scan).

Our study has limitations that need to be considered. First, we focused on cvRFs without investigating CBIs aetiology. In this context, the primary endpoint of the 2-year longitudinal SILENT study will be to identified the cause underlying CBI following a standardised etiological work up. Second, a potential diagnostic bias arises from the use of different MRI machines for brain imaging. To standardise analyses, all scans included at least DWI, T1W, T2W and FLAIR sequences permitting to evaluate the whole spectrum of CBI. Because the study reflects current imaging practices, our findings are more likely to be applicable to real-world settings. Nevertheless, some risk of CBI misdiagnosis remains, particularly with the T2W hyperintensity and T1W hypointensity phenotype. Although imaging analyses were not centralised at a single site, in both centres they were performed exclusively by neuroradiologists trained in the interpretation of such imaging and lesion types. Regarding statistical analyses, missing data were removed through analysis. Thirdly, a single measurement may be insufficient to accurately reflect long-term blood pressure control and likely overestimates HBP prevalence. Indeed, few therapeutic modifications were based on the single office measurement and mostly referred to the general practicioner for further measurements and therapeutic modifications. To mitigate this limitation, when available, HBP diagnosis was supplemented by additional assessments such as ambulatory or 24-hour monitoring, or by a secondary measurement. During follow-up, we will be able to assess the precise number of patients who have benefited from antihypertensive treatment modifications. Additionally, the lack of a standardised OSAS screening scale in routine visits should be acknowledged as a limitation. We also acknowledge the potential for collider bias, since some patients entered the cohort through brain MRI performed for cognitive symptoms; however, the majority of MRIs were for non-cognitive indications, and patients with major cognitive impairment were excluded. Finally, interpreting the number of CBI as a marker of severity is problematic, as multiple concomitant lesions may result from a proximal embolic aetiology rather than reflecting true disease burden, particularly in patients with non-lacunar infarcts.

In conclusion, our study reports that modifiable cvRFs are prevalent and often poorly controlled in patients with CBIs detected on routine neuroimaging. Additionally, we found that the presence and the number of cvRFs were associated with the CBI burden. These findings underscore the importance of specialised consultations to optimise risk factor control for primary stroke prevention. Future studies should specifically include CBI populations and investigate whether single or multiple interventions aimed at optimising cvRF profile might reduce the risk of future stroke or slow the progression of cerebrovascular damage.

## Supplementary Material

aakaf006_Supplemental_material_Figure_1_600dpi

aakaf006_Supplemental_table_1
