# Supplementary figures and images for: Cardiovascular risk factor control in patients with covert brain infarcts in the prospective SILENT cohort study
Source: Eur Stroke J. 2026 Jan 1;11(1):aakaf006. doi: 10.1093/esj/aakaf006 (PMC12866629; doi:10.1093/esj/aakaf006)

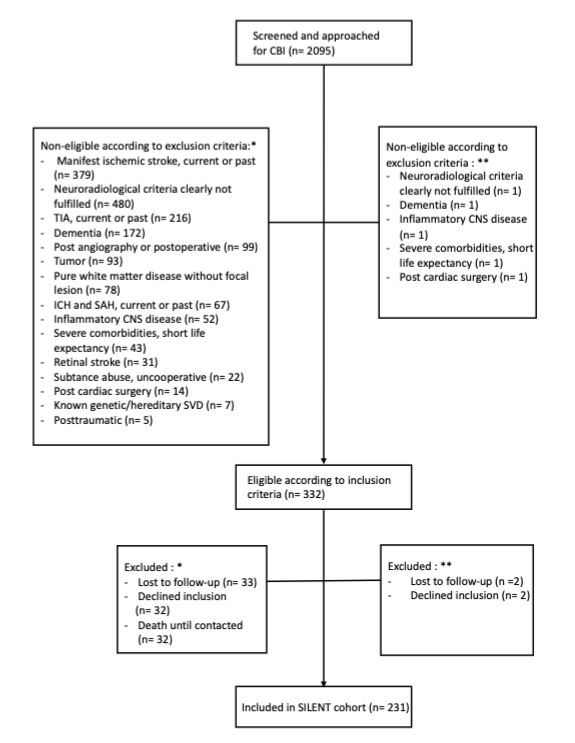

Supplement: aakaf006_Supplemental_material_Figure_1_600dpi [file aakaf006_supplemental_material_figure_1_600dpi.jpeg]
