# Supplementary material for: Cardiovascular risk factor control in patients with covert brain infarcts in the prospective SILENT cohort study
Source: Eur Stroke J. 2026 Jan 1;11(1):aakaf006. doi: 10.1093/esj/aakaf006 (PMC12866629; doi:10.1093/esj/aakaf006)
Supplement: aakaf006_Supplemental_table_1 [file aakaf006_supplemental_table_1.docx]

**Supplemental table 1*.* Comparisons of demographics and clinical characteristics between patients recruited in Bern and Tours.**

| **Demographics and clinical characteristics** | **Subtype** | **SILENT cohort Tours - N = 52 (%)** | **SILENT cohort Bern - N = 179 (%)** |
| --- | --- | --- | --- |
| **Mean age ± sd - years** |  | **63.8 ± 13.1** | **65.6 ± 13.9** |
| **Mean CBI count ± sd – number *** |  | **2.3 ± 1.5** | **1.8 ± 1.2** |
| **Male sex - no. (%)** |  | **27/52 (51.9)** | **103/179 (57.5)** |
|  |  |  |  |
| **Past medical history :** |  |  |  |
|  | *Atrial fibrillation* | **6/52 (11.5)** | **18/179 (10.1)** |
|  | *Personal CAD* | **7/52 (13.5)** | **33/174 (19)** |
|  | *Personal PAD* | **5/52 (9.6)** | **13/177 (7.3)** |
|  | *Mean CHADVASC ± sd* | **2.5 ± 1.6** | **1 ± 1** |
|  |  |  |  |
| **Cardiovascular risk factor in past medical history :** |  |  |  |
|  | *Hypertension* | **30/52 (57.7)** | **101/176 (57.4)** |
|  | *Diabetes* | **8/52 (15.4)** | **26/179 (14.5)** |
|  | *Dyslipidemia* | **22/52 (42.3)** | **105/157 (66.9)** |
|  | *OSAS* | **7/51 (13.7)** | **39/155 (25.2)** |
|  | *Tabac* | **8/52 (15.4)** | **38/177 (21.5)** |
|  |  |  |  |
| **Usual treatments :** |  |  |  |
|  | *Antiplatelet drugs* | **13/52 (25)** | **94/179 (52.5)** |
|  | *Anticoagulation drugs* | **6/52 (11.5)** | **26/179 (14.5)** |
|  | *Antihypertensive drugs* | **29/52 (55.8)** | **95/179 (53.1)** |
|  | *Statin/Ezetimibe* | **20/52 (38.5)** | **83/179 (46.4)** |
|  | *Oral Antidiabetic drugs* | **8/52 (15.4)** | **18/179 (10.1)** |
|  | *Mean glucose ± sd - g/L* | **1.1 ± 0.4** | **1.2 ± 0.5** |

*Sd : standard deviation; nb : number; OSAS : obstructive sleep apnea syndrome; HbA1c : glycated haemoglobin; LDL : low density lipoprotein; CAD : coronary artery disease; PAD : peripheral artery disease.*

**Mann–Whitney U test: p = 0.006*
